# The Effect of HIV and the Modifying Effect of Anti-Retroviral Therapy (ART) on Body Mass Index (BMI) and Blood Pressure Levels in Rural South Africa

**DOI:** 10.1371/journal.pone.0158264

**Published:** 2016-08-23

**Authors:** Andrea B. Feigl, David E. Bloom, Goodarz Danaei, Deenan Pillay, Joshua A. Salomon, Frank Tanser, Till W. Bärnighausen

**Affiliations:** 1 Harvard Medical School, Department of Global Health and Social Medicine, 641 Huntington Ave, 02115, Boston, MA, United States of America; 2 Harvard TH Chan School of Public Health, Dept. of Global Health and Population, 677 Huntington Ave, 02115, Boston, MA, United States of America; 3 Africa Health Research Institute, Somkhele, South Africa; 4 Division of Infection & Immunology, University College London, London, United Kingdom; 5 School of Nursing and Public Health, University of KwaZulu-Natal, Durban, South Africa; 6 Institute of Public Health, Heidelberg University, Heidelberg, Germany; British Columbia Centre for Excellence in HIV/AIDS, CANADA

## Abstract

**Background:**

High BMI and blood pressure are leading chronic disease risk factors in South Africa. Longterm effects of HIV and ART on adiposity and blood pressure are poorly understood, and direct comparisons of risk factor trajectories in HIV^-^ versus HIV^+^ populations are rare.

**Methods:**

In 2003 and 2010, height, weight, and blood pressure were recorded in a study population (n = 505) in KwaZulu-Natal, South Africa (30% adult HIV prevalence). We modeled change in BMI and BP longitudinally in HIV^-^ individuals (n = 315), seroconverters (n = 32), HIV^+^ patients not on ART (HIV^+^ART^−^; n = 52), HIV^+^ patients on ART for 0–<2 years as of 2010 (HIV^+^ART^0–<2 yrs^; n = 18), patients on ART for 2–5 years (HIV^+^ART^2–5yrs^; n = 44), and a subgroup with unknown HIV status (n = 44). Difference-in-differences were assessed in reference to the HIV^-^ population.

**Results:**

Between 2003 and 2010, BMI increased significantly in the HIV^-^ group, by 0.874 (95% CI 0.339, 1.41; p = 0.001), to 30.4. BMI drop was significantly greater in HIV^+^ART^0-<2yrs^ than in HIV^+^ART^2–5yrs^ (p = 0.005). DID in BMI in HIV^+^ART^0-<2yrs^ versus the reference was -5.21 (95% CI -7.53, -2.90; p = 0.001), and DID in HIV^+^ART^2–5yrs^ versus reference was -1.35 (95% CI -2.89, 0.189; p = 0.086). DID in SBP in HIV^+^ART^−^vs HIV^-^ DID was -7.55 mmHg (95% CI -13.2 to -1.90; p = 0.009).

**Conclusion:**

Short-term ART (0–<2 years) was associated with larger weight loss than either no ART or long-term ART. Once on ART for 2+ years, individuals ‘caught up’ on weight gain with the HIV^-^ population. Our results showcase the importance of health system readiness to address the burgeoning double burden of disease in South Africa.

## Introduction

South Africa, with a HIV prevalence of about 25% among 25–49 year olds [1], is the country with the largest HIV^+^ population in the world (6.4 million, out of a population of 52.3 million, in 2012 [2, 3]). In 2011, HIV/AIDS was the number one cause of years of life lost (YLL) in South Africa [4], and the 7^th^ leading cause of death overall [5]. Extensive ART rollout has been underway since 2004, and even the poorest and hardest hit communities in rural KwaZulu-Natal (South-East South Africa) provided ART to over 31% of those in need in 2011 [6].

Whilst South Africa has made major gains in its control of the HIV-epidemic [7], the chronic disease burden is increasing [8–13]. In 2010, high body mass index (BMI) and blood pressure (BP) were the top two and three risk factors contributing to the burden of disease in South Africa [4]. Cerebrovascular diseases, heart disease, and diabetes were the number three to five largest killers in 2011, respectively [5].

Although cardiovascular (CVD) risk factors affect both HIV-negative and HIV-positive populations [8, 9], little is known about the modifying effect of ART on CVD risk factors, particularly in low-income settings. A recent meta-analysis of cardio-metabolic traits in HIV-positive and HIV-negative populations in Sub-Saharan Africa (SSA) concluded that HIV infection was associated with both lower systolic and diastolic blood pressure, but evidence on the effect of ART on blood pressure was largely lacking [14]. Recent literature has revealed additional cross-sectional evidence that pre-ART weight is a predictor of onset of diabetes on ART [15], that ART is associated with non-HIV related, chronic morbidity [16], and that ART is associated with increased central fat (a cardio-metabolic disease marker) and reduced peripheral fat [17]. We were unable to identify longitudinal studies with a HIV^-^ control group and that spanned both long periods before and after ART rollout. Nonetheless, such population-level evidence is of critical importance, as it is plausible that HIV and ART affect CVD risk factors via weight gain among individuals on ART, survival benefit of ART, chronic HIV-induced inflammation, and other ART side effects.

To address this research gap, our study assessed the effect of HIV and the modifying effect of ART on body mass and blood pressure in rural KwaZulu-Natal between 2003 and 2010, with the two following advantages over similar studies in this field: 1) we include a HIV^-^ group to account for the secular trend in risk factors, and 2) the first time point pre-dates ART rollout well over one year for all participants, serving as a unique baseline measure.

## Methods

### Data source

Nested within a longitudinal, population-based HIV surveillance study, surveys on height, weight, and blood pressure were conducted both in 2003/04 and 2010 in rural uMkhanyakude in KwaZulu-Natal, South Africa, where adult HIV prevalence was close to 30% in 2010 [6]. The survey data from these two rounds are linked at the individual level. The 2003 survey antedated the large-scale rollout of ART, which started in August 2004 in this community. The 2010 survey took place against the background of widespread ART coverage of 24% [6]. The surveys are described in detail elsewhere [8, 9]. Individuals were eligible for HIV testing and weight, height and blood pressure measurement if they were residents in the Africa Center’s defined geographic surveillance area (DSA). In 2003/4, the eligible age range was 25–49 years for women, and 25–54 years for men; in 2010, the eligible age range for the HIV, BMI, and BP survey was >15 years of age.

Household membership was self-defined on the basis of links to other members [18, 19]. HIV-infected participants on ART were all enrolled at 16 primary healthcare (PHC) clinics in the Hlabisa subdistrict of uMkhanyakude. ART guidelines have indicated that treatment should be initiated at CD4 cell counts <200 cells/μl between 2004 and 2010, the timeframe of this study.

### Study population for weight and blood pressure measurements

**Fig 1:**
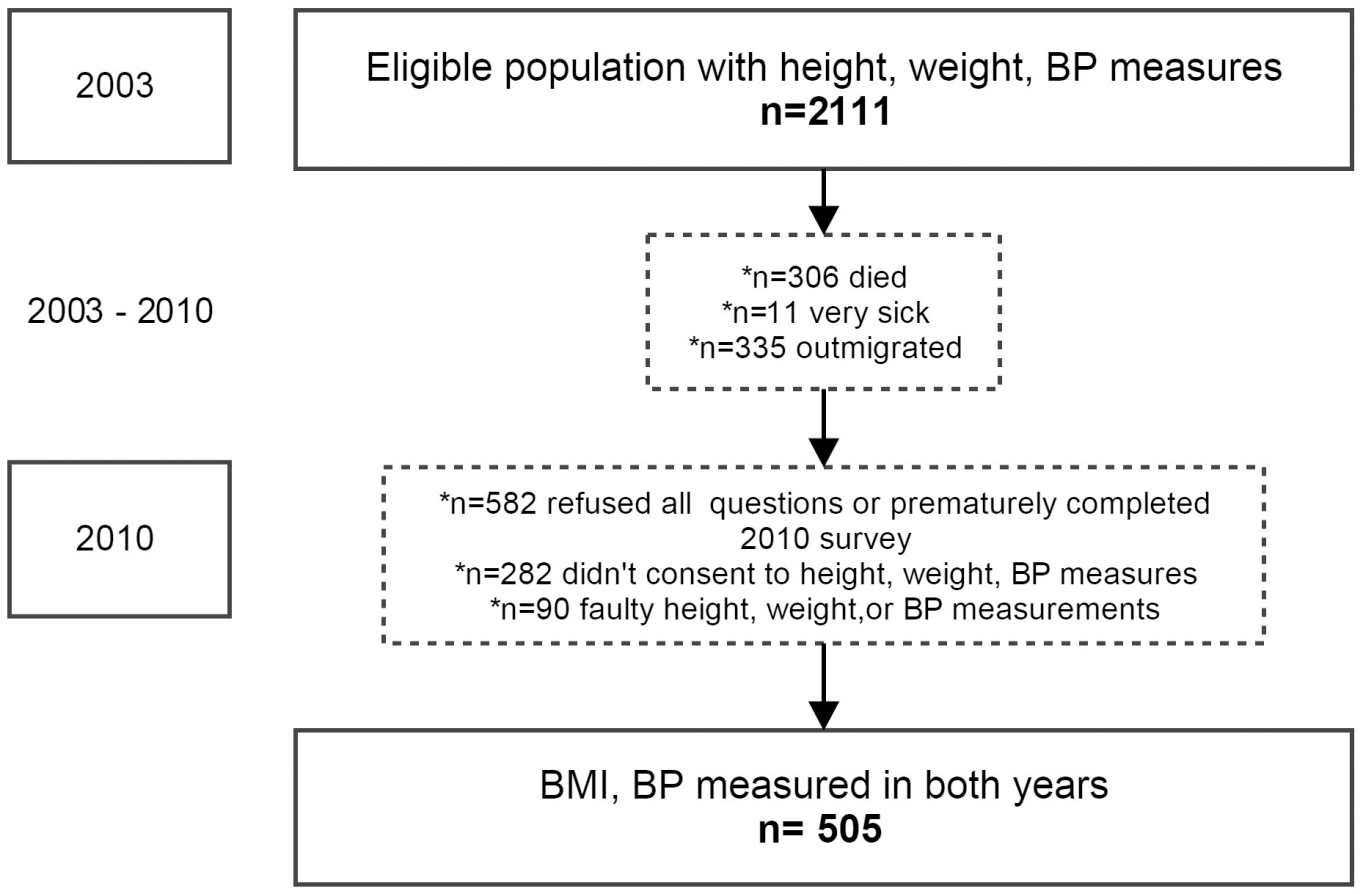
Study Population. 2111 individuals had both BMI and blood pressure measurements in 2003. This population was comprised of females 25-49yrs old and males 25-54yrs old who lived in the ACDIS catchment area, were residents, were visited by a fieldworker, and completed the full demographic survey, in addition to the WHO STEPS survey which included BMI and BP measurements. 505 individuals had survey, BMI, and BP measurements both in 2003 and 2010. These individuals constitute the main, complete-case study population. For the IPW sensitivity analyses, baseline information of all individuals except those who died and were very sick was used to calculate inverse probability weights.

In 2003, 2111 eligible individuals within the DSA completed weight, height, and blood pressure measurements as part of a WHO STEPS survey [20]. At the start of the second survey round in 2010, 306 of the original population had died, 11 persons had become very ill, and 335 had out-migrated. Of the remaining 1,459 participants, 582 refused all questions or did not fully complete the 2010 demographic survey; 283 did not consent to weight, height, and BP measurements in 2010, and 90 had faulty BMI or BP measurements. Thus, 505 individuals had survey, BMI, and BP measurements in both 2003 and 2010 (Fig 1). These individuals constitute the main, complete-case study population.

### Measurements

HIV status was assessed by enzyme-linked immunoabsorbent assay (ELISA) of EDTA anti-coagulated blood samples in the Africa Centre virology laboratory, using HIV-1/HIV-2 ELISA. Every first positive test was confirmed by a second test. Height, weight, and blood pressure were measured following the WHO STEPS protocol [20]. Blood pressure was taken three consecutive times, and the averages of the second and third measurements were used to estimate Systolic Blood Pressure (SBP) and Diastolic Blodd Pressure (DBP). Underweight was defined as a BMI <18.5 kg/m^2^, normal weight was 18.5 ≤BMI <25, and overweight and obesity as a BMI 25 to <30, and ≥30, respectively. Stage 1 hypertension was defined as SBP ≥140 mmHg and SBP <160 mmHg or DBP ≥90 mmHg, and stage 2 hypertension as SBP ≥160mm Hg or DBP ≥100 mmHg.

### Statistical Analysis

The dependent variables for all models were BMI, SBP, and DBP. The exposures of interest were HIV status and (length of) ART use. We modeled change in BMI and blood pressure over time in HIV-negative individuals, seroconverters, HIV-positive patients not on ART (HIV^+^ART^-^), HIV-positive patients on ART for 0–<2 years (HIV^+^ART^0–<2yrs^), HIV on ART for 2–5 years (HIV^+^ART^2–5^ yrs), and a subgroup with unknown HIV status. Seroconverters were defined as those HIV-negative in 2003, but HIV-positive by 2010. None of the sero-converters was on ART by 2010, since their CD4-counts exceeded the treatment cutoff. Difference-in-differences in body mass and blood pressure between 2003 and 2010 in all subgroups were assessed with reference to the HIV^-^ group. We adjusted our analyses for baseline levels of potential confounders, which were age (continuous), sex, and geographic area.

We conducted an IPW (Inverse Probability Weighting) analysis to account for changes in the distribution of population characteristics in the different comparison groups due to loss to follow up between 2003 and 2010 (2111 versus 505 participants). Baseline information on all individuals except those who died and were very sick was used to compute stabilized inverse probability-of-censoring weights (IPCWs) [21]. The IPCW method assigns a weight to each non-censored individuals and allows them to ‘represent’ those who have been lost to follow-up after adjusting for observed patient characteristics at baseline [22–24]. Censoring weights were estimated using age (continuous), sex, HIV (treatment) status, education, wealth quintile, geographic sampling area, and household assets.

We tested the robustness of the complete-case analysis with several sensitivity analyses. First, we included information on those who died and fell ill to calculate the probability weights. For censoring weights, death and severe illness are often regarded as extreme events. Characteristics of individuals who died or were severely ill might therefore not be comparable to the characteristics of those who remain in the population. However, since the study population is a high HIV prevalence population, these conventional assumptions might not hold. Therefore, we conducted a sensitivity analysis that included baseline information on those who died or fell severely ill [25, 26].

To test for the sensitivity of our results to individuals with very high or very low weight, we excluded those with BMI ≥50. As BMI might be an independent risk factor for high BP, we also adjusted for BMI at baseline in (assuming no collider bias [27]). Further IPCWs included fewer covariates to calculate the probability weights: age, sex, general health, and the assets index at baseline. The results for the effects of HIV and ART on DBP over time are presented as sensitivity analyses, since SBP and DBP are generally highly correlated. All analyses were performed using Stata 13.

Ethical approval for data collection, linkage, and analysis was obtained from the University of KwaZulu-Natal Biomedical Research Ethics Committee (BREC), and written informed consent was obtained from all participants. The analyses presented here used only secondary and anonymized data and were thus exempted from ethics review as based on a decision by the Harvard University Institutional Review Board.

## Results

### Baseline Characteristics

In 2003, the mean age of the complete-case, main study population was 39.5 (SD: 7.2) years, and mean BMI was 29.0 kg/m^2^ (SD: 7.2). 13.1% of respondents were in the highest asset index quintile, 23.6% had completed 2^ary^ or higher education, and 37.1% reported to be in very good or excellent health. Baseline SBP was 126.6 mmHg (SD: 20.2), and DBP was 80.6 mmHg (SD: 13.2) (Table 1).

**Table 1:** Baseline characteristics.

	Baseline cohort (n = 2111)	2003-only, excluding dead or very sick (n = 1289)	Died or very sick (n = 317)	Complete-case population (n = 505)
**Years of age (SD)**	**37.8 (7.3)**	**37.0 (7.1)**	**38.1 (7.7)**	**39.5 (7.2)**
HIV^-^	39.0 (7.2)	38.5 (7.3)	42.1 (7.8)	40.8 (7.0)
HIV^+^	36.0 (7.2)	35.7 (6.7)	36.6 (7.4)	38.0 (7.6)
HIV^unknown^	36.5 (7.1)	36.2 (6.9)	37.8 (7.4)	38.0 (7.0)
**Weight, kgs (SD)**	**73.8 (18.9)**	**75.3 (19.3)**	**69.3 (18.7)**	**75.0 (18.8)**
HIV^-^	74.8 (18.7)	75.3 (19.0)	71.0 (16.2)	75.9 (19.0)
HIV^+^	69.0 (17.9)	70.8 (19.5)	66.9 (18.3)	69.4 (18.4)
HIV^unknown^	75.6 (19.1)	77.0 (19.3)	71.3 (20.0)	76.1 (18.1)
**BMI (SD)**	**28.0 (7.4)**	**28.5 (7.5)**	**26.0 (7.4)**	**29.0 (7.2)**
HIV^-^	28.4 (7.4)	28.5 (7.5)	26.5 (6.5)	29.4 (7.2)
HIV^+^	26.3 (7.2)	27.0 (7.8)	25.6 (7.7)	27.1 (6.8)
HIV^unknown^	28.6 (7.4)	29.1 (7.4)	26.3 (7.6)	29.4 (7.3)
**SBP (SD)**	**124.9 (19.0)**	**124.7 (18.0)**	**123.7 (22.1)**	**126.6 (20.2)**
HIV^-^	126.9 (19.4)	126.3 (18.5)	131.9 (21.1)	127.8 (20.9)
HIV^+^	122.4 (17.8)	122.6 (15.5)	119.9 (19.7)	126.2 (20.4)
HIV^unknown^	124.1 (19.1)	124.1 (18.3)	124.1 (24.3)	125.9 (19.1)
**DBP (SD)**	**79.4 (12.5)**	**79.29 (12.0)**	**79.2 (13.7)**	**80.6 (13.2)**
HIV^-^	79.7 (12.8)	79.0 (12.3)	82.4 (13.6)	81.0 (13.5)
HIV^+^	78.6 (11.8)	79.1 (11.0)	77.6 (12.7)	79.4 (12.8)
HIV^unknown^	79.4 (12.4)	79.5 (12.0)	79.6 (14.5)	80.5 (12.9)
**Sex (female, %)**	**68.7**	**66.1**	**57.1**	**81.8**
HIV^-^	66.1	62.4	36.7	79.9
HIV^+^	68.9	69.6	62.1	75.3
HIV^unknown^	71.2	68.2	61.5	88.5
**Asset index (% in highest quintile)**	**19.1**	**22.4**	**15.8**	**13.1**
HIV^-^	16.5	18.4	18.3	15.2
HIV^+^	14.5	18.5	12.9	15.4
HIV^unknown^	24.4	27.8	18.0	23.6
**Education (% 2**^**ary**^ **or higher)**	**34.0**	**38.9**	**29.0**	**23.6**
HIV^-^	28.2	32.8	20.0	20.5
HIV^+^	31.3	35.2	30.0	24.7
HIV^unknown^	41.3	46.3	32.5	28.2
**Health (% Very good or excellent)**	**37.1**	**39.7**	**31.9**	**34.3**
HIV^-^	36.2	39.3	35.0	31.4
HIV^+^	35.2	38.8	29.3	35.3
HIV^unknown^	39.2	40.4	33.3	38.5
**HIV status (%)**				
HIV^-^	40.3	40.7	18.9	52.3
HIV^+^	20.9	17.2	44.2	16.8
HIV^unknown^	38.8	42.2	36.9	30.9

This table shows unadjusted sex, age, weight, height, BMI, SBP, DBP, HIV status, educational status, self-reported health status, and asset index characteristics of the baseline cohort population (n = 2111), as well as of sub-components of this population: those who were measured in 2003 only and neither died nor fell very sick (n = 1289), those who either died or fell very ill (n = 317), and the complete-case population (n = 505). The 2003-only population includes those who succesfully completed the 2003 survey, but outmigrated by 2010, prematurely completed the 2010 demographic survey, did not consent to biometric measurements in 2010, and had faulty biometric measurements in 2010. Note that those who died or fell very sick by 2010 are not included among the 2003-only population. Of the 317 individuals who died or became very ill between 2003 and 2010, 140 were HIV-positive, and 11 had been on ART. The complete-case population are those with full measurements in both 2003 and 2010.

We found several differences in the baseline characteristics between the ‘2003-only’ and the ‘complete-case’ population (Table 1). Most notably, the complete-case population was older, heavier, had higher blood pressure, and had a higher percentage of females. The complete-case population was also overall poorer and less educated, had lower self-reported health, and had a higher prevalence of HIV^-^ individuals (Table 1).

Further examining the baseline differences between the HIV subgroups in the complete-case population, we found that age differed significantly among the four HIV status groups, with the highest age in the HIV^-^ group (40.2 years, SD: 7.2) and the lowest age in the HIV^+^ART^-^ group (37.3 years, SD: 7.1) (S1 Table. BMI differed significantly among subgroups: it was highest in the HIV^unknown^ group (30.1kg/m^2^, SD 7.0), and lowest in the HIV^+^ART^+^ group (27.2, SD: 7.1). Among those on ART, the average time on ART was 2.5 years (SD: 1.4) between 2004 and 2010. 29% of ART users were on ART for 0-<2 years between 2004 and 2010. CD4^+^ count (measured just prior to ART initiation) was 136.5 (SD: 78.7) for all subjects who initiated ART. Gender composition, SBP, and DBP at baseline did not differ significantly between HIV-status groups (S1 Table). The minimum elapsed time between the first BMI and BP measurement and ART initiation was 1.2 years; the maximum time between these two measurements was 7.2 years (S1, S2 and S3 Figs). The average time elapsed between the first BMI and BP measurement and ART initiation in the HIV^+^ART^0–<2yrs^ group was 6.0 years (SD: 0.6), and 3.5 years (SD: 1.0) in the HIV^+^ART^2–5 yrs^ group.

### Effect of ART and HIV on change of BMI and Blood Pressure

Between 2003 and 2010, BMI increased significantly in the HIV^-^ group, by 0.874 kg/m^2^ (95% CI 0.339 to 1.41; p = 0.001), to 30.4 kg/m^2^. BMI significantly decreased by -4.34 kg/m^2^ (95% CI -6.58 to -2.10; p<0.001) in the HIV^+^ART^0-<2yrs^ population, to 25.9 kg/m^2^ in 2010. All other groups experienced non-significant changes in BMI between 2003 and 2010 (Table 2a).

**Table 2:** Effect of ART and HIV on longitudinal change of BMI.

**2a: Population average model: effect of ART and HIV on longitudinal change of BMI**								
	**BMI, 2003** (95% CI)	**BMI, 2010** (95% CI)	**ΔBMI (03–10)** (95%CI)	**p-value for first difference**	**DID effect** (95%CI)	**p-value** (DID)	**Global p-value**	**ART duration p-value**
HIV^-^	29.5 (28.7, 30.3)	30.4 (29.7, 31.1)	0.874 (0.339, 1.41)	0.001**	*(ref)*		<0.001**	
Seroconverters	28.6 (26.3, 30.9)	28.3 (26.0, 30.7)	-0.274 (-1.95, 1.41)	0.749	-1.15 (-0.252, 2.83)	0.202	<0.001**	
HIV^+^ART^-^	27.6 (25.8, 29.4)	27.4 (25.6, 29.2)	-0.196 (-1.51, 1.12)	0.771	-1.07 (-2.50, 0.361)	0.173	<0.001**	
HIV^+^ART^0–<2 yrs^	30.2 (27.1, 33.3)	25.9 (22.8, 29.0)	-4.34 (-6.58, -2.10)	<0.001**	-5.21 (-7.53, -2.89)	<0.001**	<0.001**	0.005**
HIV^+^ART^2–5 yrs^	26.5 (24.5, 28.5)	26.1 (24.0, 28.0)	-0.475 (-1.91, 0.957)	0.515	-1.35 (-2.89, 0.189)	0.086	<0.001**	0.005**
**2b: Effect of ART and HIV on longitudinal change of BMI (adjusted for loss to follow up)**								
HIV^-^	29.8 (28.8, 30.9)	30.4 (29.4, 31.4)	0.554 (-.228, 1.34)	0.165	*(ref)*		<0.001**	
Seroconverters	29.0 (27.2, 30.7)	28.8 (27.1, 30.6)	-0.128 (-1.06, 0.806)	0.788	-0.682 (-1.90, 0.536)	0.273	<0.001**	
HIV^+^ART^-^	27.8 (26.3, 29.3)	27.4 (25.4, 29.4)	-0.404 (1.48, 0.675)	0.463	-0.958 (-2.29, 0.375)	0.159	<0.001**	
HIV^+^ART^0–<2 yrs^	29.3 (26.0, 32.6)	26.2 (23.9, 28.5)	-3.11 (-5.66, -0.547)	0.017*	-3.6 (-6.32, -0.984)	0.007**	<0.001**	0.032*
HIV^+^ART^2–5 yrs^	26.8 (24.8, 28.7)	26.7 (25.1, 28.4)	-0.012 (-1.20, 1.18)	0.984	-0.566 (-1.99, 0.860)	0.437	<0.001**	0.032*

**2a: Population average model.** BMI changed significantly between 2003 and 2010 in the HIV-negative group and among ART users less than 2 years on ART. Data for people with unknown HIV status not shown. **2b: Population average model with IPW**. The results presented in this table are based on a population average linear regression model using IPW adjusting for missingness due to loss to follow up, migration, and non-consent (but not death and severe illness). The weights were based on age, sex, education, general health, and an assets index. The model controlled for age, sex, HIV, and ART status. BMI = body mass index, DID = difference-in-differences, ART = antiretroviral treatment.

* … significant at 0.05 level.

** … significant at the 0.01 level.

Relative to this change, BMI decreased by -5.21 kg/m^2^ (95% CI -7.53 to -2.90; p = 0.001) in the HIV^+^ART^0-<2yrs^ group, and by -1.35 kg/m^2^ (95% CI -2.89, 0.189; p = 0.086) in the HIV^+^ART^2–5yrs^ group. The relative changes in BMI between HIV^+^ART^0-<2yrs^ and HIV^+^ART^2–5 yrs^ were significantly different from each other, as reflected in the global p-value for ART duration (p = 0.005) (Table 2a). This attenuation of relative weight loss when individuals were on ART for two to five years, compared to 0 to <2 years, suggested a U-shaped association with long-term use of ART and BMI: Once on ART for two or more years, individuals ‘caught up’ on weight gain with the HIV^-^ reference population (See S4 Fig for plotted BMI and SBP trajectories in all subgroups).

The IPW sensitivity analysis showed qualitatively similar, quantitatively attenuated, results (Table 2b) compared to the complete-case analysis. We confirmed an increase in BMI in the HIV^-^ group by 2010, but it was statistically non-significant according to the IPW model. BMI in the HIV^+^ART^0–<2yrs^ group dropped by -3.66 kg/m^2^ (95% CI -6.32 to -0.984; p = 0.007) compared to the HIV^-^ reference group. The relative drop in BMI of the HIV^+^ART^2–5 yrs^ versus the HIV^-^ group was not statistically significant, paralleling the findings of the complete-case analysis. The drop in BMI was significantly greater among HIV^+^ART^0-<2yrs^ versus HIV+ART2–5yrs (p = 0.032).

Further sensitivity analyses (S2 Table) including the full baseline population to calculate probability weights confirmed the robustness of the complete-case results (S2 Table). Excluding individuals with BMI >50kg/m^2^, the drop in BMI in the HIV+ART0–<2yrs group was further attenuated (-2.43 kg/m^2^ (95% CI -4.21 to -0.644; p = 0.008)) (S2 Table). Notably, the change in BMI between 2003 and 2010 in the HIV- group was slightly higher at 1.03 kg/m^2^ (95% CI 0.614 to 1.45; p<0.001) compared to the analogous result of the complete-case analysis. Overall, the findings of both sensitivity analyses were qualitatively in alignment with the results of the complete-case analysis.

Further, with wide-scale ART starting in 2004, those starting in 2004/05 might have been more urgently in need than those starting on ART in 2006 and beyond. Consequently, the modifying effect of ART on BMI and BP might have been different in the ‘early starters’. We therefore conducted sensitivity analyses by excluding those on ART for four to five years (who initiated therapy between 2005 and 2007) for all models. None of the main results were changed with respect to the estimated effect size. However, the power was negatively impacted due to sample size reduction (results not shown).

### Blood pressure change 2003–2010

Between 2003 and 2010, modeled SBP based on the complete-case analysis dropped from 130.4 mmHg (95% CI 125.0 to 135.0) to 123.5 mmHg (95% CI 118.2 to 128.9) in the HIV^+^ART^-^ group (p_difference_ = 0.010). In the weighted analysis, the drop in SBP in the HIV^+^ART^2–5yrs^ group was also significant with a drop of -5.64 mmHg (95% CI -11.2 to -0.07; p = 0.047) (Table 3b). All other observed SBP changes between 2003 and 2010 were not statistically significant in both complete-case and weighted analysis. The results for the modeled changes in SBP of the weighted analysis were qualitatively similar to that of the complete-case analysis.

**Table 3:** Effect of ART and HIV on longitudinal change of SBP.

**3a: Effect of ART and HIV on longitudinal change of SBP**										
	**SBP 2003** (95% CI)	**SBP, 2010** (95% CI)		**ΔSBP (03–10)** (95%CI)	**p-value for first difference**	**DID effect** (95%CI)	**p-value** (DID)		**Global p-value**	**ART duration p-value**
HIV^-^	126.5 (124.3, 128.3)	127.0 (124.8, 129.1)		0.427 (-1.69, 2.54)	0.693	*(ref)*			0.070	
Seroconverters	123.6 (116.7,130.4)	129.1 (122.2,135.9)		5.5 (-1.17, 12.2)	0.106	5.01 (-1.94, 12.1)	0.156		0.070	
HIV^+^ART^-^	130.4 (125.0, 135.0)	123.5 (118.2, 128.9)		-6.86 (-12.1, -1.66)	0.010*	-7.55 (-13.2, -1.90)	0.009**		0.070	
HIV^+^ART^0–<2 yrs^	118.9 (109.9, 127.9)	118.9 (109.9,127.9)		0.00 (-8.76, 8.76)	1.00	-0.36 (-9.44, 8.72)	0.938		0.070	0.853
HIV^+^ART^2–5 yrs^	125.8 (120.0, 131.7)	124.8 (119.0, 130.7)		-0.977 (-6.64, 4.69)	0.735	-1.35 (-7.43, 4.73)	0.663		0.070	0.853
**3b: Effect of ART and HIV on longitudinal change of SBP (adjusted for loss to follow up)**										
HIV^-^	127.1 (124.8, 129.4)	125.6 (123.4, 127.7)	-1.53 (-3.88, 0.816)		0.201	*(ref)*			0.394	
Seroconverters	124.2 (118.9, 129.5)	128.3 (123.3,133.2)	4.06 (-1.32, 9.44)		0.139	5.60 (-.275,11.5)		0.062	0.394	
HIV^+^ART^-^	132.0 (125.5,138.5)	121.4 (115.7, 127.1)	-10.6 (-15.6, 5.66)		<0.001**	-9.09 (-14.6, -3.61)		0.001**	0.394	
HIV^+^ART^0–<2 yrs^	118.0 (111.0, 124.9)	116.3 (109.7, 123.0)	-1.62 (-9.00, 5.75)		0.666	-.093 (-7.82, 7.64)		0.981	0.394	0.0016**

**3a: Population average model.** SBP changed significantly between 2003 and 2010 among ART users less than 2 years on ART. **3b: Population average model with IPW**. The results presented in this table are based on a population average linear regression model using IPW adjusting for missingness due to loss to follow up, migration, and non-consent (but not death and severe illness). The weights were based on age, sex, education, general health, and an assets index. The model controlled for age, sex, HIV, and ART status. SBP = systolic blood pressure, DBP = diastolic blood pressure, DID = difference-in-differences, ART = antiretroviral treatment.

* … significant at 0.05 level.

** … significant at the 0.01 level.

We further investigated SBP changes relative to the HIV^-^ comparison group. The relative change in SBP in the HIV^+^ART^−^group was significant with -7.55 mm Hg (95% CI -13.2 to -1.90; p = 0.009) (Table 3a). This relative decline was greater in the weighted analysis, where the difference in SBP change between the HIV^+^ART^−^group and the control group was -9.09mm Hg (95% CI -14.6 to -3.61; p = 0.001) (Table 3b). Overall, the relative change in SBP compared to the HIV-negative reference group was not significantly different among all subgroups. The ART dose effect was also not statistically significant based on the complete-case analysis.

The results of several sensitivity analyses of the effect of HIV status on blood pressure change are presented in S3–S7 Tables. Adjusting for BMI at baseline, the results of the change in SBP were qualitatively similar to the complete-case results, and quantitatively fell between the results of the complete-case and the weighted analysis (S3 Table). Conducting a weighted analysis including information of those who died in the censoring weights, the drop in SBP in the HIV^+^ART^2–5yrs^ group was significant, similar to the results in the weighted analysis that excluded the information of the dead and very sick for weight estimation (S4 Table). The results of the sensitivity analysis that included fewer covariates were qualitatively similar to the results of the IPW analysis without the dead and very sick (S4 Table).

Overall, the effect of HIV status and ART on DBP differed from their observed effect on SBP: whereas there was a significant drop in SBP in the HIV^+^ART^-^ group, DBP significantly increased in the HIV^-^ and among seroconverters: both in the complete-case and the IPW analysis (S5 Table), DBP significantly increased by more than 4 mm Hg in the HIV^-^ group (p<0.001) between 2003 and 2010. Seroconverters showed an increase in DBP of >7 mm Hg (p = 0.001). None of the other observed DBP changes were statistically significant.

All DBP subgroup-specific relative changes compared to the HIV^-^ comparison group were non-significant based on the main model. When adjusting for BMI at baseline (S6 Table), using IPWs that included information of the full baseline population, and using IPWs based on fewer predictors (S7 Table), the results remained robust.

## Discussion

Our study is the first population-based, longitudinal analysis of the modifying effect of HIV and ART on body mass and blood pressure. Our data include a HIV^-^ reference group and spans periods before and after intensive ART rollout—two features thus far unique to chronic disease risk factor analyses in sub-Saharan HIV cohorts. Importantly, the HIV^-^ reference group allowed for an adjustment for the secular trend in BMI and BP. Further, the first BMI and BP measurement occurred well before wasting may have occurred in HIV^+^ individuals [28, 29], as the minimum difference between the first survey measurement and ART onset was well over a year (S2 and S3 Figs).

We present several novel findings. The HIV^+^ART^0-<2yrs^ group experienced a very significant decline in BMI compared to the HIV^-^ reference group. This relative decline was attenuated and no longer significant among those on ART between two and five years. Thus, our results suggest that ART allows individuals to recover toward a trend in weight gain experienced by the HIV^-^ population, without however surpassing their baseline weight.

This ‘trend toward normal weight’ has been postulated in previous studies [30]. It parallels the finding that large-scale long-term ART can lead to a return to pre-HIV workforce productivity [31], but also allays concerns that ART might lead to net weight gain in the long-term. However, while the ‘trend toward normal’ in this study population is likely a sign of health recovery on ART, the trend itself may not be beneficial to health in the long-term, because it represents a trend toward overweight and obesity. Our results thus warrant careful reflection. Health care providers need to judiciously consider when and for how long to counteract harmful weight loss in early stages of ART, and when to advise weight management in order to lessen CVD risk and other overweight and obesity-related complications. Our data provide first evidence that after two years on ART, there is no longer a significant difference in weight compared to the reference population. Future research needs to determine if, when, and in which ART patients to start nutrition and exercise interventions to curb weight gain.

If staff training and resources permitted, excessive weight gain and high blood pressure could be monitored and managed concurrently withART therapy.

However, research on the effectiveness of integration of chronic infectious and non-communicable disease services is still ambivalent. For instance, it is not clear whether such integration could weaken HIV treatment programs. In a systematic review on the impact of integrating primary healthcare services in LMICs at the point of delivery, Dudley and Garner examined the effect of integration on healthcare delivery, user views (satisfaction), and health status [32]. While adding services (i.e. adding diabetes screening to HIV treatment and care) improved the use of the added-on service, there was very little evidence that health status was improved by service integration. In some cases, integration also led to deteriorating service delivery [32]. Therefore, an important avenue of further study is to assess the effectiveness of alternative delivery models for chronic infectious and non-communicalbe diseases, including disease prevention, and to arrive at best practice recommendations in emerging economies and low-income settings.

In planning the integration of NCD prevention interventions into HIV treatment and care programs, additional indicators for risk screening should be considered, for instance, waist circumference and waist-to-hip-ratio (WTHR). There exists ample evidence that waist circumference and WTHR are much greater predictors of mortality and disease risk than BMI [33–36]. Several previous studies have shown that weight gain during ART therapy favors abdominal versus limb fat accumulation, thereby exposing someone on ART with the same BMI as a HIV-negative person to a higher CVD and mortality risk [30]. Women are particularly affected with visceral adipose tissue (VAT) accumulation as an effect of ART. Therefore, despite the fact that the herein presented study did not allow for examination of the change of WTHR between 2003 and 2010 between the various HIV subgroups, it is highly likely that the ‘return to normal’ BMI in the long-term ART exposure group was characterized by an increase in VAT, and thereby, a relative increase in CVD risk compared to the HIV^-^ population.

Regardin blood pressure, patients on ART showed BP stabilization over seven years; in contrast, HIV^+^ individuals who did not receive ART showed substantial drop in BP compared to ART users.

Notably, the HIV^+^ART^-^ group had the highest average systolic blood pressure in 2003. This indicates a potentially unhealthier lifestyle in the HIV^+^ART^-^ group compared to the HIV^-^ group. Further, compared to the HIV^-^ group, SBP in the HIV^+^ART^−^group showed a significant decline. We were unable to determine whether this overall drop in SBP from 130.4 mmHg to 123.5 mmHg was due to HIV-related side effects or due to improved clinical care (a SBP >130 mmHg is considered elevated blood pressure and is associated with a higher risk of cardiomyopathies, and a higher risk of recurrent strokes [37]). Likely, the large drop in SBP in this population might be explained by additional blood pressure monitoring and interventions following HIV diagnosis. To test this hypothesis, detailed healthcare records, a larger sample size, and additional longitudinal data points would be necessary.

### Limitations

We could not control for a range of potential time-variant confounders in our analysis due to lack of data. Important examples of these unmeasured potential confounders were detailed information on health status and the presence of other infections (particularly in HIV-positive patients), level of ART adherence, viral load information, drug resistance, and whether ART patients were on first or second line treatment regimen. Thus, we cannot rule out that the causal interpretation of our results is biased.

Furthermore, our study would have benefitted from more measurement points both between 2003 and 2010 and thereafter. This would have allowed us to better evaluate the BMI trajectory differences in the various BMI subgroups and to draw stronger conclusions with regard to the ‘back to normal weight’ hypothesis. As only a subset of the demographically surveyed population in 2003 and 2010 were assessed for body mass and blood pressure, our study and results might further lack wider external validity. Finally, our study suffered from a large loss to follow up, which we addressed by conducting IPW sensitivity analyses. Our results were qualitatively robust to these analyses, but further research with intensive follow up is needed to confirm our findings and to address to growing burden on non-communicable diseases (NCDs) in South Africa and surrounding nations.

## Conclusion

In this first population-based, longitudinal analysis of the modifying effect of HIV and ART on body mass and blood pressure we find that short-term ART (0–<2 years) was associated with a larger weight loss compared to either no ART or long-term ART. Once on ART for two or more years, individuals ‘caught up’ on weight gain with the HIV^-^ reference population. Our results emphasize the importance of health system readiness to address the burgeoning double burden of disease in sub-Saharan Africa. As emerging economies face the double burden of non-communicable and infectious diseases, further research based on cohort studies with both HIV-positive and HIV-negative populations will be needed to elucidate further the trends and treatment opportunities of multiple chronic diseases and risk factors in populations in sub-Saharan Africa.

## Transparency Declaration

The lead author (ABF) affirms that this manuscript is an honest, accurate, and transparent account of the study being reported; that no important aspects of the study have been omitted; and that any discrepancies from the study as planned have been explained.

## Supporting Information

S1 Fig Years between first weight measurement and Antiretroviral Treatment (ART) Initiation, all ART groups. (TIF)

S2 Fig Years between first weight measurement and Antiretroviral Treatment (ART) Initiation, long-term ART group. (TIF)

S3 Fig Years between first weight measurement and Antiretroviral Treatment (ART) Initiation, short-term ART group. (TIF)

S4 Fig Body Mass Index (BMI) and Systolic Blood Pressure (SBP) changes, 2003–2010. (TIF)

S1 File Research in Context. (DOCX)

S1 Table Additional baseline characteristics of the complete-case study population in 2003 (n = 505), including p-values. This table shows the unadjusted sex, age, weight, height, Body Mass Index (BMI), Systolic Blood Pressure (SBP), Diastolic Blood Pressure (DBP), and HIV status characteristics of the study population. Age, weight, and BMI differed significantly among the subgroups at baseline.
(DOCX)

S2 Table Effect of Antiretroviral Treatment (ART) and HIV on longitudinal change of Body Mass Index (BMI)–robustness checks. **S2a:** The results presented in this table are based on a population average linear regression model using Inverse Probability Weights (IPWs) adjusting for missingness due to loss to follow up, migration, and non-consent (but not death and severe illness). The weights were based on age, sex, education, general health, and an asset index. The model controlled for age, sex, HIV, and ART status. **S2b:** Same as S2a, but weights included information at baseline of those who subsequently died or fell ill. **S2c:** The results presented in this table are based on a population average model (without IPW), but excluded all outliers with BMI <50kg/m2 at baseline and follow-up.
(DOCX)

S3 Table Effect of Antiretroviral Treatment (ART) and HIV on longitudinal change of Systolic Blood Pressure (SBP). SBP changed significantly between 2003 and 2010 among ART users 0-<2 years on ART. *Adjusted for Body Mass Index at baseline*.
(DOCX)

S4 Table Effect of Antiretroviral Treatment (ART) and HIV on longitudinal change of Systolic Blood Pressure (SBP)–robustness checks. **S4a:** The results presented in this table are based on a population average linear regression model using IPW adjusting for missingness due to loss to follow up, migration, and non-consent (but not death and severe illness). The weights were based on age, sex, education, general health, and an assets index. The model controlled for age, sex, HIV, and ART status. **S4b:** Same as S4a, but weights included information at baseline of those who subsequently died or fell ill. **S4c: (population average model) with Inverse Probability Weights (IPW) (fewer covariates).** The results presented in this table are based on a population average linear regression model using IPW adjusting for missingness due to loss to follow up, migration, and non-consent. The weights were based on age, sex, and education. The model controlled for age, sex, HIV, and ART status.
(DOCX)

S5 Table Effect of Antiretroviral Treatment (ART) and HIV on longitudinal change of Diastolic Blood Pressure (DBP).S5a: Population average model. DBP changed significantly between 2003 and 2010 among ART users less than 2 years on ART. 3b: **S5b: Population average model with Inverse Probability Weights (IPW)**. The results presented in this table are based on a population average linear regression model using IPW adjusting for missingness due to loss to follow up, migration, and non-consent (but not death and severe illness). The weights were based on age, sex, education, general health, and an assets index. The model controlled for age, sex, HIV, and ART status.
(DOCX)

S6 Table Effect of Antiretroviral Treatment (ART) and HIV on longitudinal change of Diastolic Blood Pressure (DBP) (population average model). DBP changed significantly between 2003 and 2010 among ART users less than 2 years on ART. *Adjusted for Body Mass Index (BMI) at baseline*.
(DOCX)

S7 Table Effect of Antiretroviral Treatment (ART) and HIV on longitudinal change of Diastrolic Blood Pressure (DBP)–robustness checks. **S7a:** The results presented in this table are based on a population average linear regression model using IPW adjusting for missingness due to loss to follow up, migration, and non-consent (but not death and severe illness). The weights were based on age, sex, education, general health, and an assets index. The model controlled for age, sex, HIV, and ART status. **S7b:** Same as S7a, but weights included information at baseline of those who subsequently died or fell ill. **S7c: (population average model) with Inverse Probability Weights** (**IPW; fewer covariates).** The results presented in this table are based on a population average linear regression model using IPW to adjust for missingness due to loss to follow up, migration, and non-consent. The weights were based on age, sex, and education. The model controlled for age, sex, HIV, and ART status.
(DOCX)

S8 Table Estimation and characteristics of the Inverse Probability Weights (IPWs). (DOCX)
