# Supplementary figures and images for: The Effect of HIV and the Modifying Effect of Anti-Retroviral Therapy (ART) on Body Mass Index (BMI) and Blood Pressure Levels in Rural South Africa
Source: PLoS One. 2016 Aug 23;11(8):e0158264. doi: 10.1371/journal.pone.0158264 (PMC4995007; doi:10.1371/journal.pone.0158264)

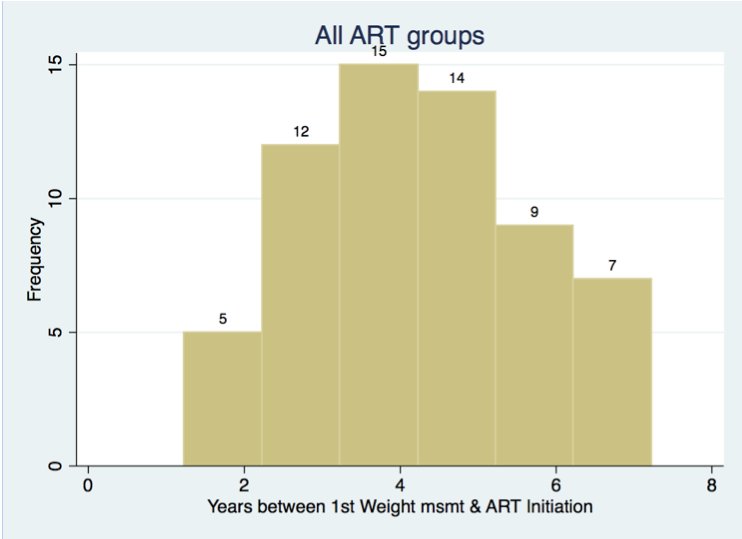

Supplement: S1 Fig — (TIF) [file pone.0158264.s001.tif]

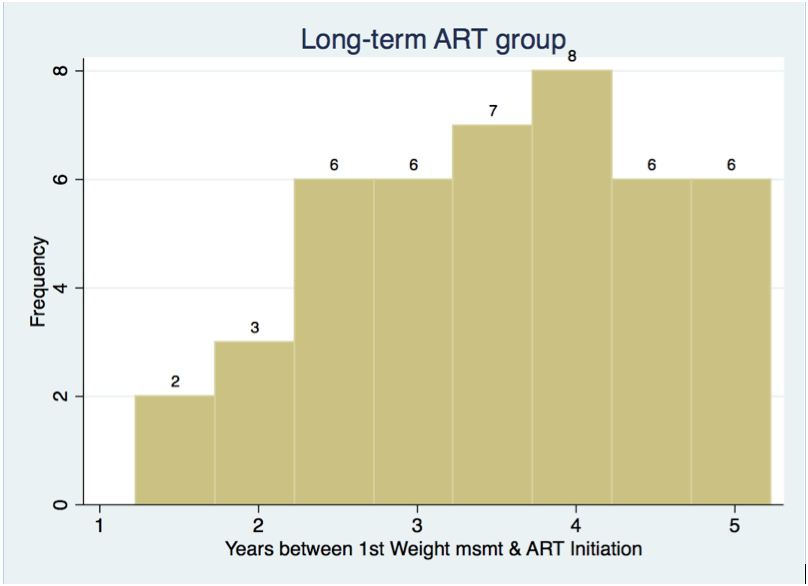

Supplement: S2 Fig — (TIF) [file pone.0158264.s002.tif]

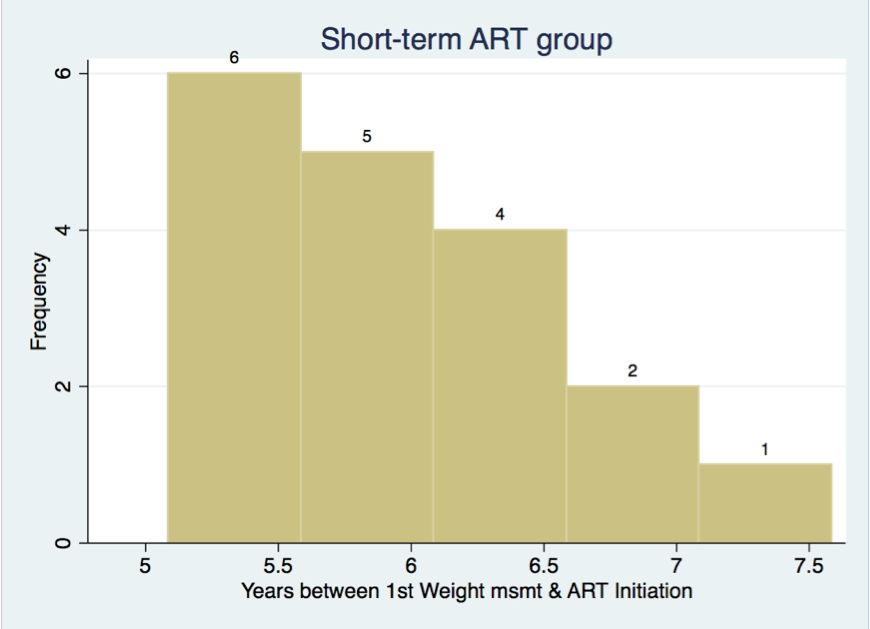

Supplement: S3 Fig — (TIF) [file pone.0158264.s003.tif]

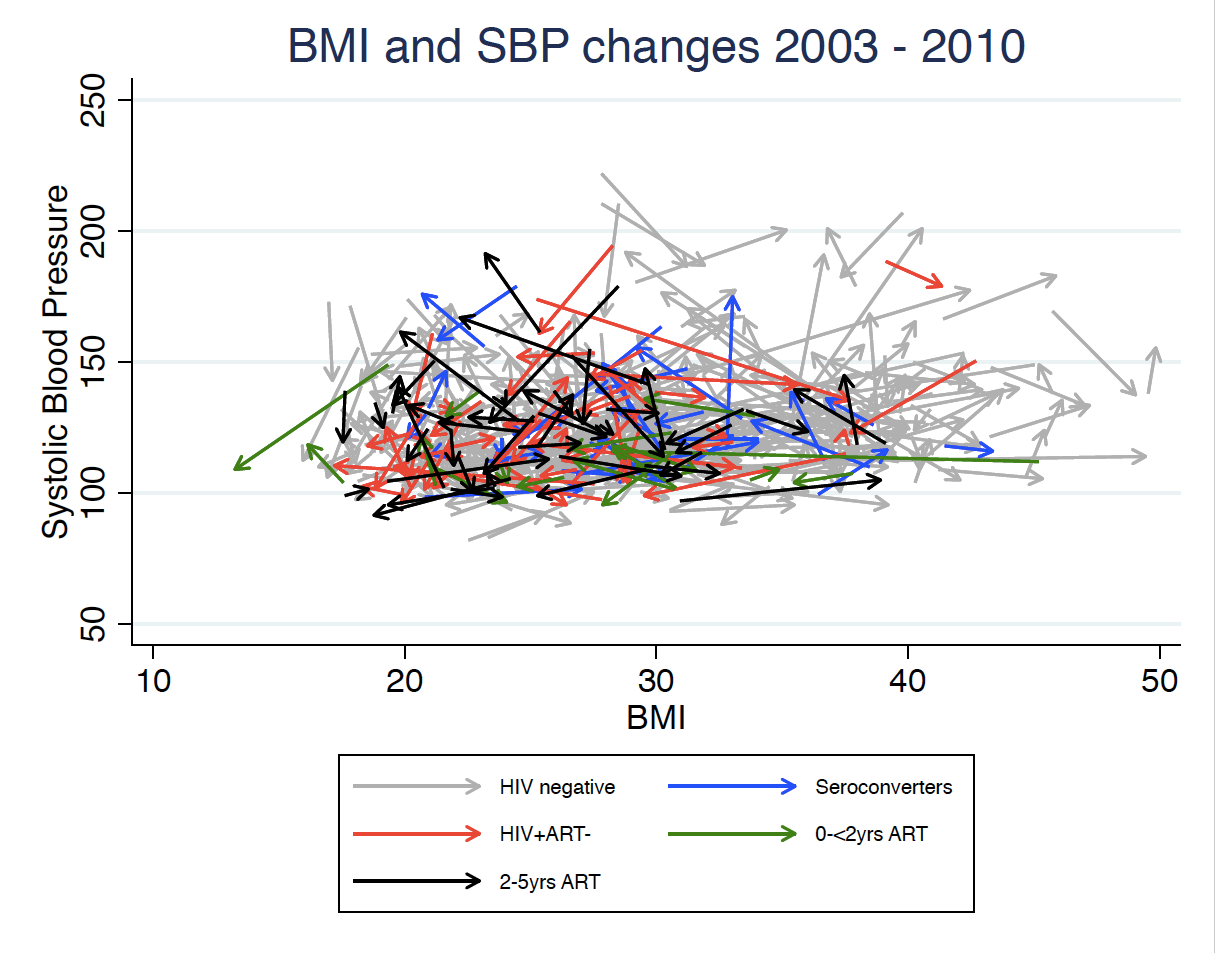

Supplement: S4 Fig — (TIF) [file pone.0158264.s004.tif]
