# Supplementary material for: The Effect of HIV and the Modifying Effect of Anti-Retroviral Therapy (ART) on Body Mass Index (BMI) and Blood Pressure Levels in Rural South Africa
Source: PLoS One. 2016 Aug 23;11(8):e0158264. doi: 10.1371/journal.pone.0158264 (PMC4995007; doi:10.1371/journal.pone.0158264)
Supplement: S1 File — (DOCX) [file pone.0158264.s005.docx]

**Research in Context**

We searched PubMed with the terms “HIV”, “ART”, “Africa”, and (“Cardiovascular” or “BMI” or “Blood pressure” or “Weight” not “Pregnancy”), for articles published between Jan 1, 2000, and November 25, 2014. We restricted our search to articles available in English. We identified a comprehensive systematic review that included all articles identified through our original search up to January 1, 2012. We then restricted our search to articles published between Jan 1, 2012, and November 25, 2014. Among the 109 identified studies, 106 studies were excluded based on title, abstract, and data review.

The 2013 meta-analysis concluded that HIV infection was associated with both lower systolic and diastolic blood pressure, but evidence on the effect of ART on blood pressure was weak or non-existent.^1^ Dillon et al emphasized the need for further research in this area to more reliably manage chronic disease risk in HIV-infected populations in SSA.

The additional three studies revealed during our review showed that pre-ART weight was a predictor of onset of diabetes on ART^2^, that ART was associated with non-HIV related, chronic morbidity^3^, and that ART was associated with increased central fat (a cardio-metabolic disease marker) and reduced peripheral fat.^4^ We did not identify studies that were longitudinal studies including both a HIV^-^ control group and that spanned both periods before and after ART rollout.

**Interpretation**

Based on our findings, short-term ART (0 to <2 years) is associated with a larger weight loss compared with no or long-term ART. This attenuation of relative weight loss when individuals were on ART for two to five years, compared to 0 to <2 years, suggests a U-shaped association with long-term use of ART and BMI: Once on ART for two or more years, individuals ‘catch up’ on weight gain with the HIV^-^ reference population. Our study revealed the need for additional evidence of the effect of HIV and ART on cardiovascular and chronic disease risk, particularly in high-prevalence, low-income populations. In addition, further evidence on optimal health systems solutions to address the double burden of chronic and infectious disease is needed.

**References**

1. Dillon DG, Gurdasani D, Riha J, et al. Association of HIV and ART with cardiometabolic traits in sub-Saharan Africa: a systematic review and meta-analysis. *International journal of epidemiology* 2013; **42**(6): 1754-71.

2. Moyo D, Tanthuma G, Mushisha O, et al. Diabetes mellitus in HIV-infected patients receiving antiretroviral therapy. *South African medical journal = Suid-Afrikaanse tydskrif vir geneeskunde* 2014; **104**(1): 37-9.

3. Mutevedzi PC, Rodger AJ, Kowal P, Nyirenda M, Newell ML. Decreased chronic morbidity but elevated HIV associated cytokine levels in HIV-infected older adults receiving HIV treatment: benefit of enhanced access to care? *PloS one* 2013; **8**(10): e77379.

4. Goedecke JH, Micklesfield LK, Levitt NS, et al. Effect of different antiretroviral drug regimens on body fat distribution of HIV-infected South African women. *AIDS research and human retroviruses* 2013; **29**(3): 557-63.
