# Supplementary material for: The Effect of HIV and the Modifying Effect of Anti-Retroviral Therapy (ART) on Body Mass Index (BMI) and Blood Pressure Levels in Rural South Africa
Source: PLoS One. 2016 Aug 23;11(8):e0158264. doi: 10.1371/journal.pone.0158264 (PMC4995007; doi:10.1371/journal.pone.0158264)
Supplement: S1 Table — This table shows the unadjusted sex, age, weight, height, Body Mass Index (BMI), Systolic Blood Pressure (SBP), Diastolic Blood Pressure (DBP), and HIV status characteristics of the study population. Age, weight, and BMI differed significantly among the subgroups at baseline. (DOCX) [file pone.0158264.s006.docx]

S1 Table: Additional baseline characteristics of the complete case study population in 2003 (n=505), including p-values.

| Characteristics mean (SD) | Overall sample population (n=505) | HIV^-^ (n=347) | HIV^+^ART^-^ (n=52) | HIV^+^ART^+^ (n=62) | Refused Testing (n=44) | p-value |
| --- | --- | --- | --- | --- | --- | --- |
| Sex (female, %) | 81.78 % | 81.27% | 80.77% | 77.42% | 93.18% | 0.23 |
| Years of age (SD) | 39.5 (7.2) | 40.2 (7.2) | 37.3 (7.1) | 37.6 (7.8) | 38.8 (5.5) | 0.005** |
| Weight, kgs (SD) | 74.9 (18.8) | 76.1 (19.1) | 69.1 (15.6) | 71.2 (19.1) | 77.44 (17.3) | 0.024* |
| Height, cms (SD) | 160.8 (7.3) | 160.9 (7.3) | 159.1 (9.1) | 162.0 (6.9) | 160.7 (5.9) | 0.226 |
| BMI (SD) | 29.0 (7.2) | 29.5 (7.4) | 27.4 (6.0) | 27.2 (7.1) | 30.1 (7.0) | 0.029* |
| Years on ART (SD) |  |  |  | 2.5 (1.4) |  |  |
| % on ART for 0-1.9 years |  |  |  | 29.03% |  |  |
| CD4+ count at ART initiation |  |  |  | 136.5 (78.7) |  |  |
| SBP, n=502, (SD) | 126.7 (20.2) | 127.1 (20.7) | 129.3 (22.2) | 122.3 (17.4) | 125.6 (17.8) | 0.270 |
| DBP, n=502, (SD) | 80.6 (13.2) | 80.9 (13.5) | 81.0 (14.0) | 78.0 (10.7) | 81.0 (12.5) | 0.436 |
