# Supplementary material for: The Effect of HIV and the Modifying Effect of Anti-Retroviral Therapy (ART) on Body Mass Index (BMI) and Blood Pressure Levels in Rural South Africa
Source: PLoS One. 2016 Aug 23;11(8):e0158264. doi: 10.1371/journal.pone.0158264 (PMC4995007; doi:10.1371/journal.pone.0158264)
Supplement: S3 Table — SBP changed significantly between 2003 and 2010 among ART users 0-<2 years on ART. Adjusted for Body Mass Index at baseline. (DOCX) [file pone.0158264.s008.docx]

S3 Table: Effect of ART and HIV on longitudinal change of SBP.

| **Population average model – adjusted for BMI at baseline** | | | | |
| --- | --- | --- | --- | --- |
| **HIV Group** | **SBP 2003 (95% CI)** | **SBP, 2010 (95% CI)** | **ΔSBP (03-10) (95%CI)** | **p-value for first difference est.** |
| HIV^-^ | 126.6  (124.4,128.8) | 126.9 (124.7,129.2) | 0.368  (-1.75, 2.48) | 0.734 |
| Seroconverters | 123.418 (116.6,130.2) | 128.82  (122.1,135.5) | 1.67  (-3.94, 7.28) | 0.560 |
| HIV^+^ART^-^ | 131.6  (126.4,136.8) | 124.4  (119.2,129.62) | -7.23  (-12.4, 2.03) | 0.006** |
| HIV^+^ART^0–<2 yrs^ | 118.3  (109.8,126.9) | 118.3  (109.8,126.9) | 0.00  (-8.77, 8.77) | 1.00 |
| HIV^+^ART^2–5 yrs^ | 127.226  (121.8, 132.6) | 126.241  (120.9,131.6) | -0.984  (-6.64, 4.67) | 0.733 |
