# Supplementary material for: The Effect of HIV and the Modifying Effect of Anti-Retroviral Therapy (ART) on Body Mass Index (BMI) and Blood Pressure Levels in Rural South Africa
Source: PLoS One. 2016 Aug 23;11(8):e0158264. doi: 10.1371/journal.pone.0158264 (PMC4995007; doi:10.1371/journal.pone.0158264)
Supplement: S4 Table — S4a: The results presented in this table are based on a population average linear regression model using IPW adjusting for missingness due to loss to follow up, migration, and non-consent (but not death and severe illness). The weights were based on age, sex, education, general health, and an assets index. The model controlled for age, sex, HIV, and ART status. S4b: Same as S4a, but weights included information at baseline of those who subsequently died or fell ill. S4c: (population average model) with Inverse Probability Weights (IPW) (fewer covariates). The results presented in this table are based on a population average linear regression model using IPW adjusting for missingness due to loss to follow up, migration, and non-consent. The weights were based on age, sex, and education. The model controlled for age, sex, HIV, and ART status. (DOCX) [file pone.0158264.s009.docx]

S4 Table: Effect of ART and HIV on longitudinal change of SBP – robustness checks.

|  | **S4a: IPW (without dead and very sick)** | | **S4b: IPW with all lost to follow-up** | | **S4c: IPW without dead and very sick, fewer covariates** | |  |
| --- | --- | --- | --- | --- | --- | --- | --- |
|  | **ΔSBP (03-10) (95%CI)** | **p-value** | **ΔSBP (03-10) (95%CI)** | **p-value** | **ΔSBP (03-10) (95%CI)** | **p-value** | |
| **HIV^-^** | -1.53 (-3.88, 0.816) | 0.201 | -1.72 (-4.10, 0.661) | 0.157 | -1.12 (-3.39, 1.14) | 0.330 | |
| **Seroconverters** | 4.06 (-1.32, 9.44) | 0.139 | 4.13 (-1.22, 9.48) | 0.130 | 4.04 (-0.938, 9.75) | 0.106 | |
| **HIV^+^ART^-^** | -10.6 (-15.6, 5.66) | <0.001** | -11.4 (-16.5, -6.17) | <0.001** | -9.81 (-14.7, -4.90) | <0.001** | |
| HIV^+^ART^0–<2 yrs^ | -1.62 (-9.00, 5.75) | 0.666 | -1.98 (-10.7, 6.79) | 0.659 | -.595 (-7.74, 6.55) | 0.870 | |
| HIV^+^ART^2–5 yrs^ | -5.64 (-11.2, -0.070) | 0.047* | -6.04 (-11.7, -0.387) | 0.036* | -4.84 (-10.3, 0.628) | 0.083 | |
