# Supplementary material for: The Effect of HIV and the Modifying Effect of Anti-Retroviral Therapy (ART) on Body Mass Index (BMI) and Blood Pressure Levels in Rural South Africa
Source: PLoS One. 2016 Aug 23;11(8):e0158264. doi: 10.1371/journal.pone.0158264 (PMC4995007; doi:10.1371/journal.pone.0158264)
Supplement: S5 Table — DBP changed significantly between 2003 and 2010 among ART users less than 2 years on ART. 3b: S5b: Population average model with Inverse Probability Weights (IPW). The results presented in this table are based on a population average linear regression model using IPW adjusting for missingness due to loss to follow up, migration, and non-consent (but not death and severe illness). The weights were based on age, sex, education, general health, and an assets index. The model controlled for age, sex, HIV, and ART status. (DOCX) [file pone.0158264.s010.docx]

S5 Table: Effect of ART and HIV on longitudinal change of DBP. S5a: Population average model.

| **S5a: Effect of ART and HIV on longitudinal change of DBP** | | | | | | | | |
| --- | --- | --- | --- | --- | --- | --- | --- | --- |
|  | **DBP 2003 (95% CI)** | **DBP, 2010 (95% CI)** | **ΔDBP (03-10) (95%CI)** | **p-value for first difference** | **DID effect (95%CI)** | **p-value (DID)** | **Global p-value** | **ART duration p-value** |
| HIV^-^ | 80.6  (79.2, 82.0) | 85.4  (84.0, 86.9) | 4.85  (3.42, 6.28) | <0.001** | *(ref)* | | 0.735 |  |
| Seroconverters | 78.9  (74.5, 83.4) | 86.9  (82.5, 91.4) | 8.00  (3.48, 12.5) | 0.001** | 3.15  (-1.59, 7.89) | 0.192 |  |  |
| HIV^+^ART^-^ | 81.5  (78.0, 85.0) | 84.7  (81.2, 88.2) | 3.15  (-.374, 6.67) | 0.080 | -1.70  (-5.50, 2.10) | 0.380 |  |  |
| HIV^+^ART^0–<2 yrs^ | 77.0  (71.1, 82.8) | 80.0  (74.2, 85.9) | 3.06  (-2.87, 8.98) | 0.312 | -1.79  (-7.89, 4.30) | 0.564 |  | 0.0683 |
| HIV^+^ART^2–5 yrs^ | 79.7  (76.0, 83.5) | 79.7  (77.8, 85.4) | 1.84  (-2.00, 5.67) | 0.348 | -3.01  (-7.11, 1.08) | 0.149 |  |  |
| **S5b: Effect of ART and HIV on longitudinal change of DBP (adjusted for loss to follow up)** | | | | | | | | |
| HIV^-^ | 80.8  (79.2, 82.5) | 85.0  (83.6, 86.4) | 4.19  (2.57, 5.82) | <0.001** | *(ref)* | | 0.284 |  |
| Seroconverters | 78.5  (74.6, 82. 3) | 86.2  (81.7, 90.7) | 7.76  (3.18, 12.3) | 0.001** | 3.56  (-1.29, 8.43) | 0.150 |  |  |
| HIV^+^ART^-^ | 81.5  (76.6, 86.4) | 83.9  (80.0, 87.8) | 2.36  (-0.882, 5.61) | 0.153 | -1.83  (-5.46, 1.80) | 0.324 |  |  |
| HIV^+^ART^0–<2 yrs^ | 76.03  (71.0, 81.0) | 78.8  (74.4, 83.3) | 2.79  (-2.22, 7.80) | 0.275 | -1.40  (-6.67, 3.86) | 0.602 |  | 0.0092** |
| HIV^+^ART^2–5 yrs^ | 80.8  (77.2, 84.3) | 80.1  (77.1, 83.0) | -0.691  (-4.62, 3.24) | 0.730 | -4.88  (-9.13, -0.631) | 0.024* |  |  |
