# Supplementary material for: The Effect of HIV and the Modifying Effect of Anti-Retroviral Therapy (ART) on Body Mass Index (BMI) and Blood Pressure Levels in Rural South Africa
Source: PLoS One. 2016 Aug 23;11(8):e0158264. doi: 10.1371/journal.pone.0158264 (PMC4995007; doi:10.1371/journal.pone.0158264)
Supplement: S6 Table — DBP changed significantly between 2003 and 2010 among ART users less than 2 years on ART. Adjusted for Body Mass Index (BMI) at baseline. (DOCX) [file pone.0158264.s011.docx]

S6 Table: Effect of ART and HIV on longitudinal change of DBP (population average model).

| **Population average model – adjusted for BMI at baseline** | | | | | |
| --- | --- | --- | --- | --- | --- |
| **HIV Group** | **DBP 2003 (95% CI)** | **DBP, 2010 (95% CI)** | **ΔDBP (03-10) (95%CI)** | **p-value for first difference est.** |  |
| HIV^-^ | 80.6  (79.2, 82.0) | 85.4  (84.0,86.8) | 4.79  (3.36, 6.22) | <0.001** |  |
| Seroconverters | 78.7  (74.4, 83.0) | 86.6  (82.4, 90.8) | 7.86  (3.35,12.4) | 0.001** |  |
| HIV^+^ART^-^ | 82.5  (79.3, 85.7) | 85.3  (82.1, 88.6) | 2.85  (-.670, 6.36) | 0.113 |  |
| HIV^+^ART^0–<2 yrs^ | 76.7  (71.4, 82.0) | 79.8  (74.5, 85.1) | 3.06  (-2.88, 8.99) | 0.313 |  |
| HIV^+^ART^2–5 yrs^ | 80.8  (77.3, 84.3) | 82.6  (79.2, 86.1) | 1.82  (-2.00, 5.65) | 0.350 |  |
