# Supplementary material for: The Effect of HIV and the Modifying Effect of Anti-Retroviral Therapy (ART) on Body Mass Index (BMI) and Blood Pressure Levels in Rural South Africa
Source: PLoS One. 2016 Aug 23;11(8):e0158264. doi: 10.1371/journal.pone.0158264 (PMC4995007; doi:10.1371/journal.pone.0158264)
Supplement: S8 Table — (DOCX) [file pone.0158264.s013.docx]

**S8 Table: Estimation and characteristics of the IPWs**

|  | **Estimated Inverse Probability Weights** | |
| --- | --- | --- |
| **Weight Specification** | **Mean (SD)** | **Min/ Max** |
| 1. Probability of participation predicted based on geographic sampling block, HIV status (at baseline), age, sex, self reported health status (at baseline), highest education attained (at baseline), and asset index (at baseline); missing variables were coded via missing indicators; those lost to follow up due to death or severe illness were not included | 0.999 (0.674) | 0.357 / 5.92 |
| 2. Probability of participation predicted based on geographic sampling block, HIV status (at baseline), age, sex, self reported health status (at baseline); missing variables were coded via missing indicators; those lost to follow up due to death or severe illness were not included | 1.01 (0.532) | 0.364 / 3.85 |
| As in 1., and those lost to follow up due to death or severe illness were included | 1.01 (0.738) | 0.314 / 7.29 |
